# Feasibility and acceptability of a technology-based, rural weight management intervention in older adults with obesity

**DOI:** 10.1186/s12877-020-01978-x

**Published:** 2021-01-12

**Authors:** John A. Batsis, Curtis L. Petersen, Matthew M. Clark, Summer B. Cook, David Kotz, Tyler L. Gooding, Meredith N. Roderka, Rima I. Al-Nimr, Dawna Pidgeon, Ann Haedrich, K. C. Wright, Christina Aquila, Todd A. Mackenzie

**Affiliations:** 1 Division of Geriatric Medicine, School of Medicine, and Department of Nutrition, Gillings School of Global Public Health, University of North Carolina at Chapel Hill, 5017 Old Clinic Building, Chapel Hill, NC 27599 USA; 2 Dartmouth-Hitchcock, Geisel School of Medicine, and The Dartmouth Institute for Health Policy, Hanover, NH USA; 3 Dartmouth College, Hanover, NH USA; 4 Mayo Clinic Rochester, Department of Psychiatry and Psychology, and Division of Endocrinology, Rochester, MN USA; 5 University of New Hampshire, Durham, NH USA

**Keywords:** Weight, Telehealth, Disparities

## Abstract

**Background:**

Older adults with obesity residing in rural areas have reduced access to weight management programs. We determined the feasibility, acceptability and preliminary outcomes of an integrated technology-based health promotion intervention in rural-living, older adults using remote monitoring and synchronous video-based technology.

**Methods:**

A 6-month, non-randomized, non-blinded, single-arm study was conducted from October 2018 to May 2020 at a community-based aging center of adults aged ≥65 years with a body mass index (BMI) ≥30 kg/m^2^. Weekly dietitian visits focusing on behavior therapy and caloric restriction and twice-weekly physical therapist-led group strength, flexibility and balance training classes were delivered using video-conferencing to participants in their homes. Participants used a Fitbit Alta HR for remote monitoring with data feedback provided by the interventionists. An aerobic activity prescription was provided and monitored.

**Results:**

Mean age was 72.9±3.9 years (82% female). Baseline anthropometric measures of weight, BMI, and waist circumference were 97.8±16.3 kg, 36.5±5.2 kg/m^2^, and 115.5±13.0 cm, respectively. A total of 142 participants were screened (*n*=27 ineligible), and 53 consented. There were nine dropouts (17%). Overall satisfaction with the trial (4.7+ 0.6, scale: 1 (low) to 5 (high)) and with Fitbit (4.2+ 0.9) were high. Fitbit was worn an average of 81.7±19.3% of intervention days. In completers, mean weight loss was 4.6±3.5 kg or 4.7±3.5% (*p*< 0.001). Physical function measures of 30-s sit-to-stand repetitions increased from 13.5±5.7 to 16.7±5.9 (p< 0.001), 6-min walk improved by 42.0±77.3 m (*p*=0.005) but no differences were observed in gait speed or grip strength. Subjective measures of late-life function improved (3.4±4.7 points, *p*< 0.001).

**Conclusions:**

A technology-based obesity intervention is feasible and acceptable to older adults with obesity and may lead to weight loss and improved physical function.

**Clinical trial registration:**

Registered on Clinicaltrials.gov #NCT03104205. Registered on April 7, 2017. First participant enrolled on October 1st, 2018.

**Supplementary Information:**

The online version contains supplementary material available at 10.1186/s12877-020-01978-x.

## Background

Obesity rates in older adults have surpassed 35% [1] of the population and have been associated with a two- to three-fold higher risk of functional decline, [2] a 30% higher risk of institutionalization [3] and mortality, [4] and $1496 annualized higher health costs compared to healthy weight older adults [5]. Caloric restriction with aerobic and resistance training is central to weight loss and improving physical function, and leads to better quality of life [6]. Efficacy trials in frail, older adults have found that diet-exercise interventions were safe, improved function, reversed frailty, [7] improved gait speed, and delayed disability [8]. Yet, weight loss in older adults is not highly endorsed by clinicians, in part due to the conflicting literature of the safety of weight loss in older adults due to the obesity paradox [9]. Busy clinicians also do not have the time nor expertise to focus on weight loss efforts [10, 11]. In fact, in Medicare beneficiaries, data showed low uptake despite coverage for obesity therapy, showing a need for new delivery systems [12].

Engaging in health promotion efforts requires proximity and frequent visits to medical facilities. This is often impossible for the 10 million older rural Americans whose obesity rates are 3.6–7% higher, physical activity levels are 7% lower, and diet quality is lower, compared to urban residents [13]. Weight and nutrition are key priorities in Rural Healthy People 2020 [14] as rural care is affected by lower healthcare access, [15] a complex natural and built environment, [14] healthcare provider workforce shortages, and a lack of rural-specific programs [16]. Barriers also include a need for social connectedness, access, and program availability [17]. Previous weight loss efforts in rural areas generally have been achieved through in-person interventions, phone calls, community-health workers, or peer coaching [18–21]. However, such health promotion efforts have rarely targeted this at-risk population which has significant co-morbidity and where obesity can markedly impair physical function [22, 23].

Self-monitoring strategies, such as completing food records or exercise logs, predict initial and sustained weight loss, [24] but may be difficult to achieve in this population. Telehealth—including telemedicine (live, two-way, video-conferencing) or remote monitoring via wearable devices (providing bi-directional, synchronous, or asynchronous data feedback)—is a delivery strategy that addresses rural health barriers by overcoming the sparsity of resources, lack of available initiatives, and workforce shortages [25]. While evidence exists in pediatric and young adult populations, [26] it is unclear whether weight-management interventions for older rural-living adults using behavioral and engagement strategies are feasible to achieve the desired outcomes. As older adults’ use of technology grows [27], using technology-based strategies can potentially be be used for clinical care. For instance, recent reviews have demonstrated that video-conferencing is feasible, acceptable and can effectively be used in older adults [28], as can fitness devices such as Fitbit for remote monitoring [29]. We designed a technology-based weight management intervention for rural older adults with obesity and evaluated its feasibility, acceptability and preliminary outcomes.

## Methods

### Study design and setting

This technology-based weight management intervention was a six-month, single-armed, weight-management intervention for older adults with obesity residing in rural New Hampshire and Vermont. Primary and secondary outcomes were evaluated at baseline 0, 2, 4, and 6 months. The study was conducted from October 2018 to May 2020 with participants continually recruited. All testing activities were conducted on-site at the local Center for Health and Aging, a community-based resource center affiliated with Dartmouth-Hitchcock. The study was approved by the Dartmouth-Hitchcock Institutional Review Board. The trial was also registered on clinicaltrials.gov under NCT#03104205.

### Study participants

Participants were recruited from physician referral. Posters and tear-off cards were delivered to offices for distribution, and the senior author (JAB) presented the study to the local clinicians. There was a maximum of 10 participants per intervention group (led by one registered dietitian nutrition (RDN) and one licensed physical therapist [PT] at any one time). The electronic medical record (EMR) was used to assess selection criteria. Participants consisted of English-speaking community-dwelling older adults aged 65+ with a BMI > 30 kg/m^2^ that had access to high-speed Internet at home. Participants were excluded if they fulfilled any of the following EMR-listed diagnoses: end-stage congestive heart failure, renal insufficiency, dementia, or hepatic failure; a terminal/life-threatening illness; severe, uncontrolled psychiatric diagnosis; nursing home or hospital admission within the past 6 month; weight loss surgery; a life-expectancy < 6 months; on obesogenic medications; or > 5% weight loss in the past 6 months. A validated Callahan screen [30] for cognitive impairment, and the validated Older Americans Resources and Services questionnaire [31] for activities of daily living were administered by phone. Scores of > 3 and > 6, respectively, fulfilled eligibility criteria. Written permission from a primary care physician was required for participation. All participants came to the community-based Center for Health and Aging for consent and study assessments. Adverse events were monitored and documented on safety sheets.

### Study intervention

**Fig. 1:**
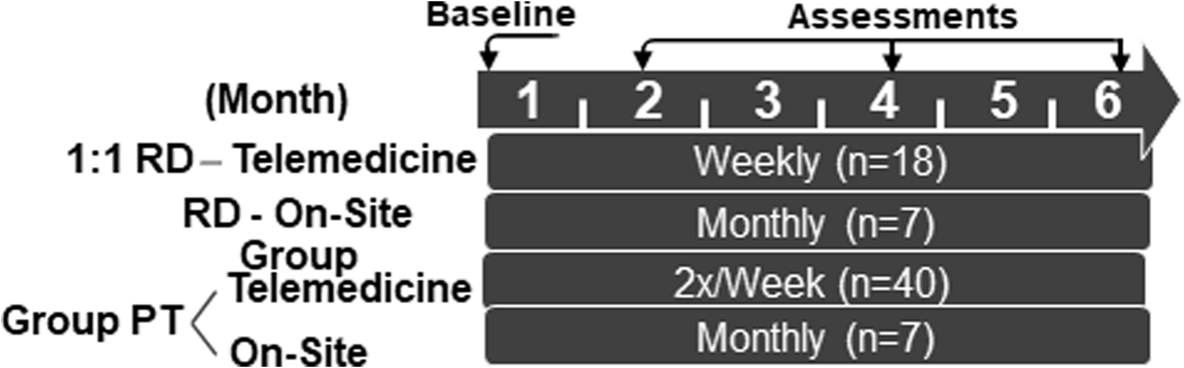
Schematic of the Components of the Technology-Based Intervention

Participants enrolled in a 26-week weight management program consisting of nutrition and exercise sessions delivered using a blend of synchronous, video-conferencing sessions with real-time communications and the use of remote monitoring using Fitbit and enhanced by periodic face-to-face interactions (Fig. 1). The nutrition sessions occurred either before or after the PT sessions. The intervention itself was based on structural elements of the social cognitive theory [32] and the technology acceptance model [33].

A RDN delivered 18 individual, 1:1 live video-conferencing nutrition sessions lasting 30 min, and 7 on-site hourly group sessions (remotely if necessary) focusing on caloric restriction (500–750 kCal/day deficit, minimum 1200 kCal/day), vitamin D (1,000 IU/day), protein intake (1–1.2 g/kg/day or 20% intake). Balanced, evidence-based individualized meal plans were guided by the Harris-Benedict equation [34] and indirect calorimetry (REEVue, Korr Medical). Motivational interviewing, goal-setting and behavioral strategies were used with written patient education materials throughout the intervention. Group on-site sessions summarized content and provided an opportunity for social connectedness. Weekly food records were reviewed, and attendance was monitored.

All study participants engaged in 75-min, twice-weekly, synchronous video-conferencing, group exercise sessions were led by a trained physical-therapist (PT) amounting to a total of 40 sessions. Over the course of the study, every 3–4 weeks, there was an on-site group session to promote social engagement amongst participants and interventionists, for a total of 7 additional sessions. The intensity of this program paralleled that of the American College of Sports Medicine recommendations for exercise in older adults [35]. Personalized resistance, flexibility and balance exercise plans were developed for all participants and conducted during each of these on-site and video-conferencing sessions. Resistance training [36] used adjustable weights and bands targeting major muscle groups (30–45 min; 8–12 reps; 2 sets), increasing loads after 15 reps of full range of motion. Flexibility exercises included static stretches (15–30 min; 30–60s each). Balance training focused on agility and coordination, and included static, dynamic, and vestibular exercises (15–30 min) [37]. Participants were also trained and guided to perform resistance, flexibility and balance exercise once weekly outside the study sessions (75 min). Progress was assessed and recorded by the physical therapist, monitored remotely, aimed at gradual workload increases for resistance training (Borg perceived exertion rate of 12 [somewhat hard]) [38]. Participants were advised to conduct the type of exercise, repetitions and sets during this specific time period. Each had a one-on-one ‘video check-in’ session weekly with the PT lasting 5 min during the intervention to adjust the proposed exercise plan and to assess progress in improving physical activity and function. Outside the structured exercise sessions, participants were guided to adopt a program of 150 min/week of moderate-intensity aerobic walking, in a minimum of 10-min bouts again, guided by the PT. [36] Hence, the total duration of study-related activities – aerobic, resistance, flexibility, balance – amounted to 375 min per week [36, 37, 39].

### Video Conferencing & Remote Monitoring

Synchronous, real-time communication with audio/video-conferencing was delivered used a HIPAA compliant version of Zoom. The RDN used a webcam-enabled laptop to conduct the 1:1 participant sessions. The PT delivered the exercise sessions in an office space, with their laptop connected to a 50″ television that permitted interaction and exercise delivery on a larger screen. A Logitech webcam and a wireless USB-microphone were used for video and audio. A Samsung Galaxy A Tab. 10.1″ tablet was given to participants. Written, step-by-step, picture-based instructions permitting them to connect the tablet to their home Wi-Fi and was then ‘mirrored’ to their personal television at home using an Amazon Firestick. At an orientation session, the research assistant demonstrated the setup; if connection issues persisted at home, they guided participants by phone. Tablet security was guided by institutional practices. Each participant was provided a Fitbit ALTA HR (Fitbit Inc., San Francisco, CA) to physical activity engagement throughout the six-month study. The research assistant demonstrated how to use and charge the device, and provided instructions on how to visualize data through the tablet-installed Fitbit-based app. Data was synchronized to a third-party software, Fitabase, which permitted data aggregation to the minute-level. Information was coded using a unique study identifier.

### Outcome measurements

Our primary outcome measures were feasibility and acceptability of the intervention. Feasibility of our recruitment criteria (screening and enrollment), intervention completion rates, and attendance rates were assessed. Our target enrollment was 48 participants which assumed an estimate of a 20% dropout rate at 6-months and an attendance rate of > 75%. We based these values on slightly more conservative estimates than our previous pilot study and other efficacy-based trials or reviews [7, 19, 40–43]. Adherence of Fitbit consisted of obtaining > 75% of available data [44] with at least 8 h of use [45]. Participant satisfaction surveys were conducted at home using RedCAP, a secure, web-based application that supports data capture for research (Additional file 1: Appendix 1). Acceptability was assessed using self-report surveys, measured at study conclusion (range 1–5, low to high). Acceptability of each measure was considered successful if the measure exceeded 4 of 5 points (> 80%).

Baseline sessions were conducted by the research assistant and consisted of two technology training components (30 min each), two National Cancer Institute Automated Self-Administered-24 dietary assessment tool evaluations (30 min each) [46] and objective physical function assessments (45 min each - see below). An initial PT assessment permitted individualization of the exercise plan by gaining insights into their baseline performance status (45 min), The co-primary effectiveness outcomes included changes in weight and in the 30-s sit-to-stand test (30STS). Weight was assessed using a standardized A+D digital scale without shoes, jackets or heavy clothing and height was measured using a stadiometer. A 5% change in weight is considered clinically significant [47]. The 30STS is a clinical construct of physical function that predicts falls and disability, is sensitive to change, and is highly correlated with quadriceps and leg press strength (test-retest reliability, r=0.89 in community dwelling older adults), leg performance (r=0.78) and 6-min walk (r=0.53, in pulmonary patients) [48–51]. Participants sat in a chair with a back, arms folded, and stood up/sat down as many times as they could for 30-s. A two-repetition increase is considered clinically significant [52]. Grip strength was assessed using a JAMAR handheld dynamometer, measured in both hands three times, alternating every 30 s; maximum values were used in the analysis. Grip strength relates to upper and lower extremity strength, and predicts mobility disability (test-retest reliability, r=0.954 in healthy elders) [53]. A clinically significant change in grip strength is 5 kg [53]. As a surrogate for submaximal exercise capacity, a 6-min walk in a long, 70 m corridor was conducted (test-retest, r=0.95, in older adults without significant disease) [54]. A change in 30 m is considered clinically significant in older adults with multimorbidity [55]. Subjective measures of physical function was assessed using the 32-item function component of the Late-Life Function and Disability Instrument (LLFDI) [56]. This measure correlates with gait speed and lower-limb function. Neither participants nor research staff were blinded to the objective outcome assessments.

### Statistical analysis

Descriptive statistics evaluated feasibility and acceptability measures. Continuous variables are represented as means ± standard deviation, and categorical values as counts (percent). An unpaired t-test or chi-square testing assessed differences between completers vs. dropouts (participants that did not complete the intervention). Intra-group comparisons of baseline and week 26 values were assessed using a paired t-test (or its non-parametric equivalent). Mixed-effects models (with a fixed effect of participant) evaluated longitudinal changes in weight, 30STS, 6-min walk and LLFDI adjusting for age and sex. We also evaluated the differences in objective physical function measures in participants losing ≥5% weight loss over time. Wear time was calculated using methods previously described [57]. All analyses were conducted using STATA v.15 or R (www.r-project.org) v.3.6. A *p*-value < 0.05 was considered statistically significant.

## Results

Participant flow is presented in Additional file 1: Appendix 1. The eligibility rate was 81%, of which 53.9% declined participation. Of the 53 participants that enrolled (46.1%), 44 (83.0%) completed the intervention. Attendance rates for both the video and on-site visits were 77 and 78.2% for the physical therapy exercise sessions respectively, and 84 and 90.0%, for the RDN-based visits respectively. Participants wore the Fitbit for an average of 81.7% of the days of the intervention and obtained an average of 4078±3819 steps per day. The mean wear time was 8.3±3.8 h per day. During the last 2 months of the intervention, there were 7 participants where we were unable to capture data due to synchronization issues. Three of these seven were also unable to synchronize their devices during months 3 or 4 as well. There were no differences in the number of steps over time (*p*=0.83).

Satisfaction rates (Table 1) were high for both the overall intervention and for specific item-based questions related to the video-conferencing components. Participants were supportive of both the virtual-based physical therapy and dietary components of the intervention. Satisfaction related to the Fitbit was slightly lower than that of the video-conferencing.

**Table 1:** Baseline Characteristics of the Telemedicine Cohort

	Overall	Completers	Dropouts	P-value
	***N***=53	***N***=44	***N***=9	
**Age, years**	72.9 ± 3.9	73.2 ± 3.9	71.4 ± 3.8	0.20
**Female Sex**	37 (69.8)	32 (72.7)	5 (55.6)	0.30
**Education**				0.17
High school	7 (13.2)	7 (15.9)	0	
Some College	15 (28.3)	14 (31.8)	1 (11.1)	
College Degree	15 (28.3)	12 (27.3)	3 (33.3)	
Post-College Degree	16 (30.2)	11 (25.0)	5 (55.6)	
**Income**				0.45
Less than $25,000	10 (18.9)	9 (20.5)	1 (11.1)	
$25,000 to $49,999	10 (18.9)	7 (15.9)	3 (33.3)	
$50,000 to $74,999	11 (20.8)	11 (25.0)	0	
$75,000 to $99,999	13 (24.5)	10 (22.7)	3 (33.3)	
$100,000 to $199,999	8 (15.1)	6 (13.6)	2 (22.2)	
$200,000 or more	1 (1.9)	1 (2.3)	0	
**Insurance**				
Medicaid	1 (1.9)	0	1 (11.1)	0.15
Medicare	48 (90.6)	41 (93.2)	7 (77.8)	0.03
Private	32 (60.4)	25 (56.8)	7 (77.8)	0.24
**Smoking Status**				0.78
Current	1 (1.92)	1 (2.3)	0	
Former	21 (40.4)	17 (38.6)	4 (50.0)	
Never	30 (57.7)	26 (59.1)	4 (50.0)	
**Marital Status**				0.53
Married	35 (66.0)	28 (63.6)	7 (77.8)	
Widow	5 (9.4)	5 (11.4)	0	
Single	13 (24.5)	11 (25.0)	2 (22.2)	
**Distance to Center, miles**	22.7 ± 19.3	24.0 ± 20.3	15.9 ± 11.1	0.25
**Distance to Center, minutes**	29.6 ± 20.6	31.1 ± 21.9	22.3 ± 10.4	0.25
**Co-Morbidities**				
Anxiety	5 (9.4)	4 (9.0)	1 (11.1)	0.85
COPD	4 (7.5)	3 (6.8)	1 (11.1)	0.66
Depression	12 (22.6)	12 (27.3)	0	0.08
Diabetes	14 (26.4)	14 (31.8)	0	0.05
Fibromyalgia	2 (3.8)	2 (4.6)	0	0.51
High Cholesterol	19 (39.9)	15 (34.1)	4 (44.4)	0.56
Hypertension	38 (71.7)	31 (70.5)	7 (77.8)	0.66
Osteoarthritis	19 (35.9)	16 (36.4)	3 (33.3)	0.86
Sleep Apnea	21 (39.6)	18 (40.9)	3 (33.3)	0.67
Stroke	2 (3.8)	1 (2.3)	1 (11.1)	0.21

All values represented are means ± standard deviation or counts (%). P-value represents difference between completers and participants that dropped out from the 6-month intervention

**Fig. 2:**
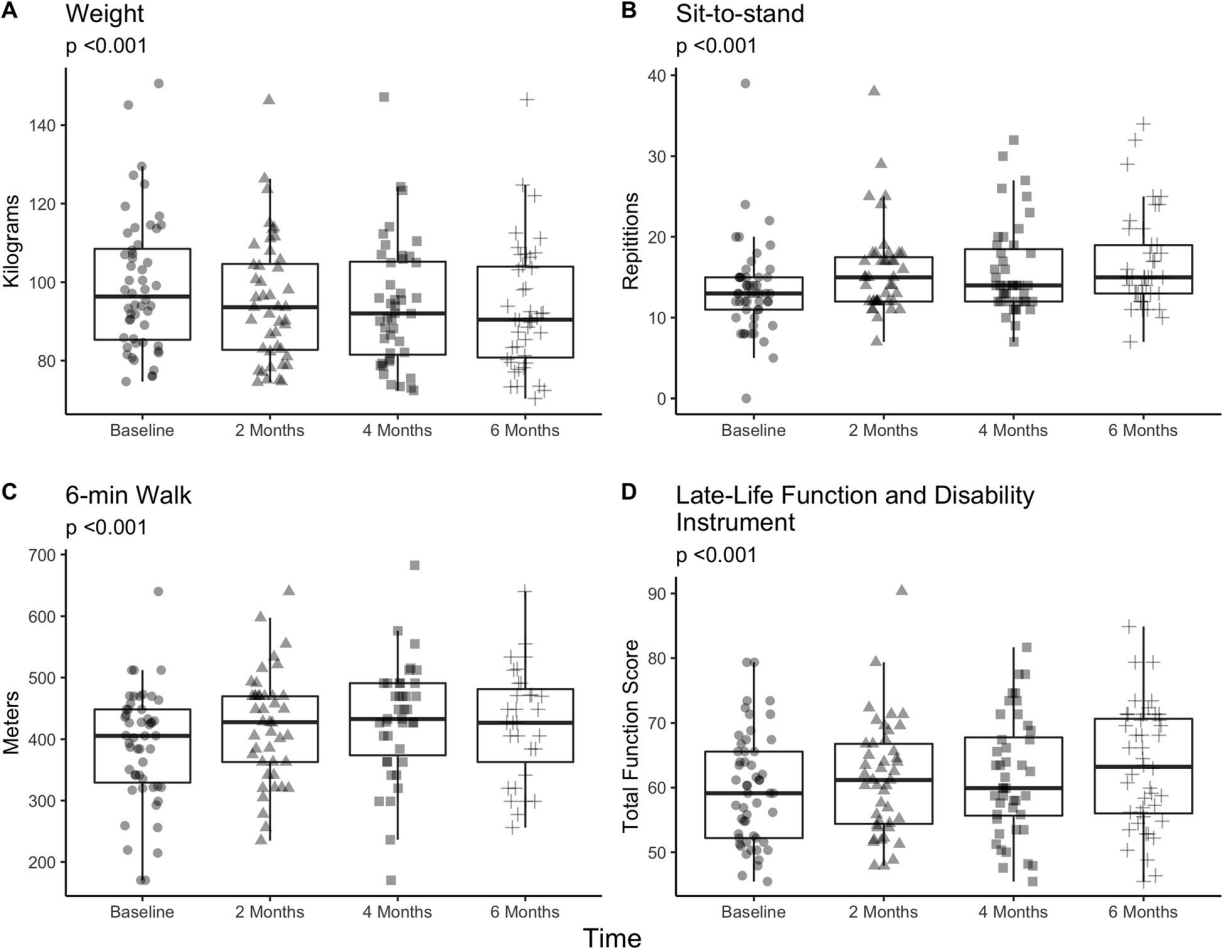
Change in Measures over 6-month intervention. Box-plots representing longitudinal changes in weight (**a**), 30-s sit-to-stand test (**b**), 6-min walk test (**c**) and total function component of Late-life function and disability index (**d**) in a Technology-Based Weight Loss Intervention in Older Adults

**Fig. 3:**
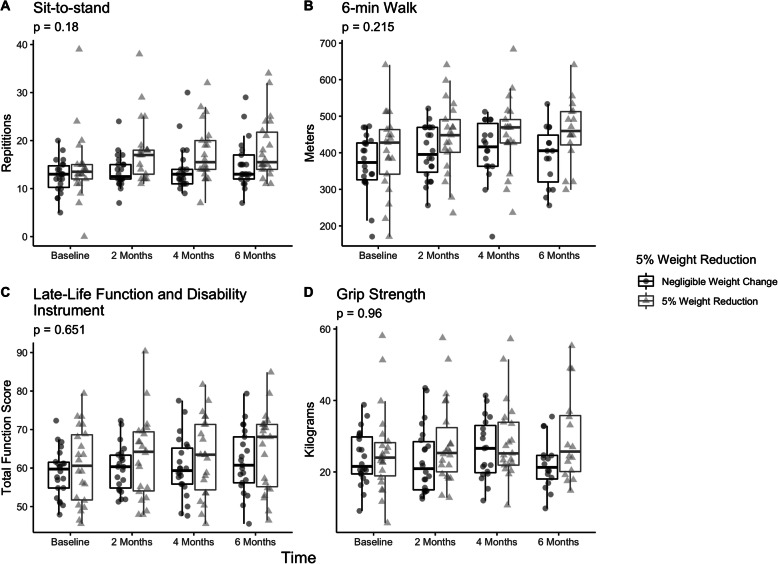
Changes in Key Measures in Participants with and without 5% Weight Loss. Box-plots representing longitudinal changes in 30-s sit-to-stand test (**a**), 6-min walk test (**b**) and function component of Late-life function and disability index (**c**) and grip strength (**d**) in a Technology-Based Weight Loss Intervention in Older Adults with and without 5% weight loss

There were no significant differences in baseline demographic characteristics in dropouts as compared to completers. Characteristics are outlined in Table 2. The mean distance and time for participants to the center was 22.7±19.3 miles and 24.0±20.3 min in enrollees. Table 3 outlines the anthropometric and objective outcome measures. Mean weight loss was 4.6±3.5 kg (4.7%), and 30STS improved from 13.5±5.7 to 16.7±5.9 repetitions (*p*< 0.001) over six-months. Fifty-percent of the cohort had clinically significant improvements in weight (*n*=22), 73% had at least a 2-repetition improvement in 30STS, and 41% had at least a 30 m improvement in 6-min walk. Changes in 6-min walk were clinically significant 42.0±77.3 m (*P*=0.005). Gait speed and grip strength did not change. Subjective measures of LLFDI also noted improvements in total, upper, basic lower, and advanced lower extremity function (p< 0.001). Graphical representation and mixed-effect models are presented in Fig. 2 and absolute values are presented in Additional file 3: Appendix #3. Participants kept on losing weight, improved their 30STS times, and improved their LLFDI scores across the different time points, while waist circumference and other functional measures plateaued earlier. Individuals losing ≥5% of their weight (pre/post) had significantly improved measures of objective physical function as compared to those not losing weight (Fig. 3). Our sensitivity analysis comparing the 33 participants with full follow-up data as compared to those without full data demonstrating differences in hyperlipidemia as a baseline co-morbidity (14 [42.4%] vs. 1 [11%], *p*< 0.001). There were no differences in primary outcomes of weight change or sit-to-stand repetitions (data not shown).

**Table 2:** Satisfaction Responses with the Technology-Based Intervention

Overall Intervention	Mean	Range
Overall Satisfaction	4.7 ± 0.6	3–5
Recommend the technology-based intervention to a family member	5.0 ± 0.2	4–5
Helpful for patients living in rural areas	4.9 ± 0.4	3–5
Helpful in assisting in achieving goals	4.7 ± 0.7	3–5
Beneficial and worth your time	4.8 ± 0.6	3–5
**Video-Based Satisfaction Measures**	**Mean**	**Range**
Satisfaction with video-conferencing device	4.4 ± 1.0	2–5
Video-conferencing assist in achieving goals	4.7 ± 0.6	3–5
Video easy to use without much difficulty	4.5 ± 0.7	3–5
Physical Therapy	**Mean**	**Range**
Program Delivery was useful	4.7 ± 0.6	3–5
Length of session	4.8 ± 0.5	3–5
Number of Sessions	4.7 ± 0.5	3–5
Nutrition	**Mean**	**Range**
Program Delivery was useful	4.9 ± 0.3	4–5
Length of session	4.9 ± 0.3	4–5
Number of Session	4.8 ± 0.5	3–5
**Willingness for Remote Intervention**	**N (%)**	**N (%)**
Physical therapy	37 (84.1)	–
Dietitian sessions	40 (90.9)	–
**Location**	**Mean**	**Range**
Easier to perform activity	4.1 ± 0.9	3–5
Adequate support for Fitbit	4.2 ± 1.0	1–5
**Satisfaction Questions on Fitbit**	**Mean**	**Range**
Overall satisfaction with Fitbit^a^ (n=2)	4.2 ± 0.9	2–5
Easy to use without much difficulty	4.3 ± 0.9	2–5
Real-time feedback helpful in promoting physical activity	4.0 ± 1.0	2–5
Helpful in achieving your goal	3.8 ± 1.0	1–5

^a^**Table range is represented as “min - max”**

**Table 3:** Preliminary Outcome Measures of Completers (n=44)

	Baseline (N=44)	Week 26 (N=44)	Difference (N=44)	Percent Change	***p*** value
**Anthropometric**					
Weight, kg	97.8 ± 16.3	93.2 ± 15.8	−4.6 ± 3.5	−4.7 ± 3.5	< 0.001
BMI, kg/m^2^	36.5 ± 5.2	34.7 ± 5.4	−1.8 ± 1.4	−5.1 ± 4.1	< 0.001
Waist circumference, cm	115.5 ± 13.0	112.8 ± 11.9	−2.6 ± 5.4	−2.1 ± 4.7	0.01
Waist to hip ratio	0.926 ± 0.081	0.935 ± 0.075	0.009 ± 0.0421	1.1 ± 4.7	0.21
**Objective Measures**					
30-s Sit To Stand, repetitions	13.5 ± 5.7	16.7 ± 5.9	3.1 ± 4.2	26.1 ± 35.6	< 0.001
^a^6 min walk test, m	387.0 ± 94.9	425.0 ± 91.7	42.0 ± 77.3	15.1 ± 28.2	0.005
^a^Gait Speed, sec	1.05 ± 0.23	1.04 ± 0.20	−0.34 ± 0.14	−1.378 ± 11.6	0.16
^a^Grip Strength, kg	24.8 ± 9.9	25.9 ± 10.6	1.2 ± 7.0	14.7 ± 51.4	0.33
**Subjective Measures**					
Late-Life Functionality					
Total	59.8 ± 8.2	63.2 ± 9.3	3.4 ± 4.7	–	< 0.001
Upper extremity	78.4 ± 13.4	81.0 ± 12.7	2.6 ± 7.7	–	< 0.001
Basic lower extremity	72.9 ± 12.5	79.3 ± 14.6	6.4 ± 11.1	–	< 0.001
Advanced lower extremity	48.7 ± 12.3	53.3 ± 14.5	4.6 ± 8.2	–	< 0.001
**Fitbit Activity Measures**	**Mean**	**Range**	**Median**		
% days worn^*^	81.7 ± 19.3	35.1–100	89.1	–	–
Steps per day	4078± 3819	0–29,884	3443	–	–

^a^Incomplete objective data on adults unable to perform follow-up assessments

Worn is defined as recorded ≥100 steps that day

There was one serious adverse event consisting of an emergency room episode for newly diagnosed atrial fibrillation. Adverse events that were definitely related to the study predominantly consisted of musculoskeletal complaints (*n*=6), skin rash due to Fitbit (*n*=1), hypoglycemia (n=1), and upset stomach (n=1) (Additional file 4: Appendix #4).

## Discussion

The findings from this technology-based intervention are timely in that it provides preliminary feasibility, acceptability, and outcomes of technology-based strategies for older adults with obesity residing in rural areas. The intervention not only led to improvements in weight, but also demonstrated key improvements in physical function measures such as 30STS and 6-min walk, both key markers of disability and independence. Notably, this intervention was conducted prior to the pandemic of COVID-19; hence, it is plausible that this technology-based intervention would have even greater appeal to an at-risk older adult population.

Our approach provided informative feasibility data that could be helpful in structuring a large-scale intervention. First, we provide that older adults can engage in the use of remote monitoring and video-conferencing, dispelling major misconceptions that this group has difficulty in using technology [58]. In fact, acceptability was high and none of the nine participants that dropped out was because of the technology itself. Second, our recruitment strategies demonstrated that our inclusion/exclusion criteria were appropriate in that < 20% were ineligible. Evaluating the potential factors that precluded involvement showed that there continues to be a slight digital divide in internet accessibility; a future trial should provide internet access to prospective participants (e.g., through a Wi-Fi hotspot). All the other negative screening criteria were aligned with medical appropriateness for weight loss in a high-risk population with medical conditions. Third, those eligible who declined (e.g., family caregiving, worry about using technology, having other commitments, or had no interest) cited the common issues faced by recruiting older adults in clinical research [59]. Caregiving efforts often impair health promotion efforts [60], and our findings confirm such observations. The program’s intensity and number of sessions may be a factor, suggesting that a future trial should reduce the number of sessions. Participants felt supported by the technical support, and that our procedures allowed engagement and monitoring throughout the study.

Recruitment of older adults with multiple chronic conditions is often challenging and hence they are under-represented in clinical trials. In fact, the National Institute of Health’s *Inclusion of the Lifespan policy* [61] promotes older adult’s participation in research. While our study focused on older adults, our efforts were successful in a short period of time as recruitment occurred within a span of 1 year. Our retention rates were favorable and aligned with those of other obesity trials [7, 40]. We recognize that participants may have been a motivated group willing to engage in health promotion efforts; those enrolled had goals of enhancing one’s health. Yet, we do caution that sampling issues should be considered when interpreting our findings as this technology-based intervention may not be feasible in those not motivated or those with additional mobility impairments.

The adherence rates in this intervention were high, both using the remote monitoring device but also with our video-visits, suggesting that rural barriers to care delivery may potentially be overcome using our approach. A major shortcoming was our inability to synchronize Fitbit device data remotely; this could only occur during the on-site, in-person sessions. Participants had issues with the Fitbit application, which occasionally logged out and did not pair up with their Fitbit device; to our knowledge, this was not a Wi-Fi issue. This is encouraging as future devices have demonstrated better (and easier) connectivity to different platforms and a future study should address such connectivity issues. As such, this was a lost opportunity for capturing data and engaging participants. Our survey findings suggested a greater needed for continuous feedback to enhance engagement efforts [29]. Future studies should consider the ease of synchronization both for participants, but also for study personnel, remotely.

Our staff was trained to assess adverse events using remote delivery, and repeatedly communicated with primary-care physicians. The number of musculoskeletal-related events was high; none were classified as serious. Hypoglycemia occurred in one participant; none experienced undue coronary events nor hypotension, all potential consequences of weight loss. Future studies should continue to involve participant’s primary-care providers, considering the high-risk population we are targeting, as they have knowledge of their medical history and can easily address medication changes as a result of weight loss.

Evidence-based strategies for video-conferencing and remote monitoring in a rural-living older populations are clearly lacking. A systematic review previously published by our group found a dearth of clinical trials; however, in those included, telemedicine could enhance older adults’ outcomes despite a high degree of trial bias [28]. Telemedicine may lead to higher weight loss [62] and may be cost-effective [63]. Yet, a separate systematic review found that telemedicine-delivered interventions reduce BMI, only one focused on older adults (IDEATel) [64]. TeleMOVE (in Veterans) found higher weight loss (vs. controls or in-person), [65] but was not focused on older adults. A six-month telemedicine study in cardiac rehabilitation noted higher weight loss and patient activation [66]. Telemedicine can also be useful for weight maintenance, and is feasible in persons at risk for falls, sarcopenia, those planning bariatric surgery, and those with sensory impairments [67–70]. Usability issues may impact the use of telemedicine; hence the need for a platform that can be navigated easily with technical support [71]. In addition to the recent need to use alternative delivery systems due to the coronavirus pandemic, our findings provide timely data on the acceptability of an intervention that limits person-to-person contact. The implications of considering such an intervention on a larger scale in a time when social distancing and isolation are rampant amongst older adults [72] cannot be understated. In fact, this proposed technology-based intervention can be easily delivered in the midst of the crisis our society is facing to maintain and preserve function through health promotion efforts.

As older adults’ use of technology grows (in 2019, Internet use was > 70%, smartphone use > 53%), using wearable technology such as Fitbit can feasibly and practically be used as clinical tools [27]. Such monitoring is promising and may elicit diet-exercise behavior change by improving self-management, tracking, social support, and goal setting; yet, results in younger populations are mixed and short-term [73, 74]. The IDEA trial found lower weight loss in the mHealth arm (vs. standard), in contrast to Spring’s trial of health coaching and digital assistants [75, 76]. Using wearables with coaching in older adults with peripheral artery disease [77] did not improve 6-min walk distance. While systematic reviews advise using mHealth in obesity trials, [78] it may be useful to couple mHealth with other modalities, including video-conferencing, to enhance care over in-person delivery [79].

The preliminary outcome findings near the threshold for clinically significant weight loss [47]. The statistically significant improvements in 30STS and 6-min walk test distances are clinically relevant. These improvements are known to be related to improved quality of life, physical function and are related to lower mortality. We recognize that this intervention may not necessarily be suitable for certain seniors who may be uncomfortable with technology or who do not have the necessary readiness to change to participate. There may be unknown biological factors that limit the intervention’s effectiveness. Future studies need to better evaluate the characteristics of individuals enrolling in this intervention to provide a personalized approach to treatment.

This study is not without limitations. This was a non-randomized, feasibility study without a control group with repeated measures at four time points strengthened our internal validity and enabling our ability to make an inference on change over time. Second, there was little ethnic diversity in a predominantly female cohort. Third, other rural areas may have different broadband capabilities or access to healthcare settings have different sociodemographic characteristics, thus may not be representative of rural dwellers. The counties served in rural New Hampshire had > 96% availability of internet connectivity [80]. Fourth, follow-up grip strength, gait speed, and 6-min walk were not obtained in 11 participants (25% of completers) due to the COVID-19 pandemic. Our sensitivity analysis compared both baseline characteristics and outcomes of weight change and 30STS finding no differences suggesting we would have similar results. Fifth, the Fitbit used is now obsolete; emerging technologies have enhanced accuracy, precision and usability. Sixth, the study’s intensity was generally high but its individual components (nutrition and exercise), align with other community-based interventions. Future research should identify the necessary intensity to achieve weight loss and improved physical function, as limiting the number of sessions could enhance longer-term intervention compliance. Last, challenges in Fitbit synchronization observed in the last 2 months of our study reduced the reliability of our step counts.

Ongoing rural weight loss programs focus mainly on behavior or nutrition, rather than including exercise, and do not target seniors. MOVE-UP’s use of community health workers delivering a diet and self-guided exercise intervention in older adults may provide insights into rural geriatric obesity care [20]. Issues related to geographic isolation among underserved, rural older adults using technology are not being addressed in these trials. Our proposed intervention potentially can contribute to rural health care-delivery science of innovative, effective, and pragmatic health-promoting activities that overcome barriers to rural healthcare in this population by improving physical function. Future trials are poised to address service gaps by using technology – and emerging telehealth regulations may permit billing, irrespective of site. Cost-effectiveness and economic analysis studies could be beneficial and helpful in the future. Telehealth is increasingly possible in older adults, with over 73–85% having access to broadband. The use of novel and practical technologies may permit integration of this technology-based intervention into health-care systems and clinical practice to ultimately improve quality and provide a scalable opportunity for widespread dissemination in rural America.

## Conclusions

This technology-based, video-monitoring and remote monitoring intervention is feasible, acceptable, and demonstrates favorable outcomes by overcoming the limitations of existing geriatric weight-loss trials, overcoming a need for proximity to medical facilities.

## Supplementary Information

**Additional file 1.** Appendix 1: CONSORT Table

**Additional file 2.** Appendix 2: Consort Diagram of Participant Flow

**Additional file 3.** Appendix 3: Adverse Events

**Additional file 4.** Appendix 4: Preliminary Outcome Measures of All Participants.

## Data Availability

The datasets generated and/or analyzed during the current study are not publicly available due to data use/privacy issues but are available from the corresponding author on reasonable request.

## Abbreviations

BMI: Body mass index
30STS: 30-s sit-to-stand
EMR: Electronic medical record
LLFDI: Late-life function and disability instrument
PT: Physical therapist
RDN: Registered dietitian nutritionist
