# Supplementary material for: Feasibility and acceptability of a technology-based, rural weight management intervention in older adults with obesity
Source: BMC Geriatr. 2021 Jan 12;21:44. doi: 10.1186/s12877-020-01978-x (PMC7801868; doi:10.1186/s12877-020-01978-x)
Supplement: Supplementary file 1 — Additional file 1. Appendix 1: CONSORT Table [file 12877_2020_1978_MOESM1_ESM.docx]

**Appendix 1 - Participant Satisfaction/Acceptability Survey Questions: (range 1-5, low to high)**

1. How would you rate your level of **Satisfaction** with the overall intervention itself?
2. Would you **recommend** this (technology-based) intervention to a family member or friend who has a weight problem?
3. Do you feel that participating via Teleconferencing is helpful for patients that live in rural areas?
4. Was the Teleconferencing interface helpful in achieving your goal?
5. Did you feel that the overall intervention was beneficial and worth your time?
6. How would you rate your level of **Satisfaction** with the Teleconference device?
7. How **Helpful** was Teleconferencing in assisting you to achieve your goals?
8. Did you find the Teleconferencing **easy to use** without much difficulty?
9. How would you rate your level of **Satisfaction** with the **physical therapist** (related to the telemedicine program – root question)
10. How would you rate your level of **Satisfaction** with the length of the video-based physical therapy sessions?
11. How would you rate your level of **Satisfaction** with the number of video-based physical sessions
12. How would you rate your level of **Satisfaction** with the **physical therapist** (related to the telemedicine program – root question)
13. How would you rate your level of **Satisfaction** with the length of the video-based dietitian sessions?
14. How would you rate your level of **Satisfaction** with the number of video-based dietitian sessions?
15. Which do you prefer more – in person or group video Physical Therapy Sessions
16. Which do you prefer more – in person group based or individual video-based Dietitian Sessions
17. Which location would it be easier for you to perform your physical activity?
18. Did you feel that you had adequate support to use and/or ask questions in relation to this device?
19. How would you rate your overall level of **Satisfaction** with the **Fitbit** device?
20. Did you find **Fitbit easy to use** without much difficulty?
21. Was **Fitbit real-time feedback helpful** in promoting physical activity
22. Was **Fitbit interface** helpful in achieving your goal?
