# Supplementary figures and images for: Feasibility and acceptability of a technology-based, rural weight management intervention in older adults with obesity
Source: BMC Geriatr. 2021 Jan 12;21:44. doi: 10.1186/s12877-020-01978-x (PMC7801868; doi:10.1186/s12877-020-01978-x)

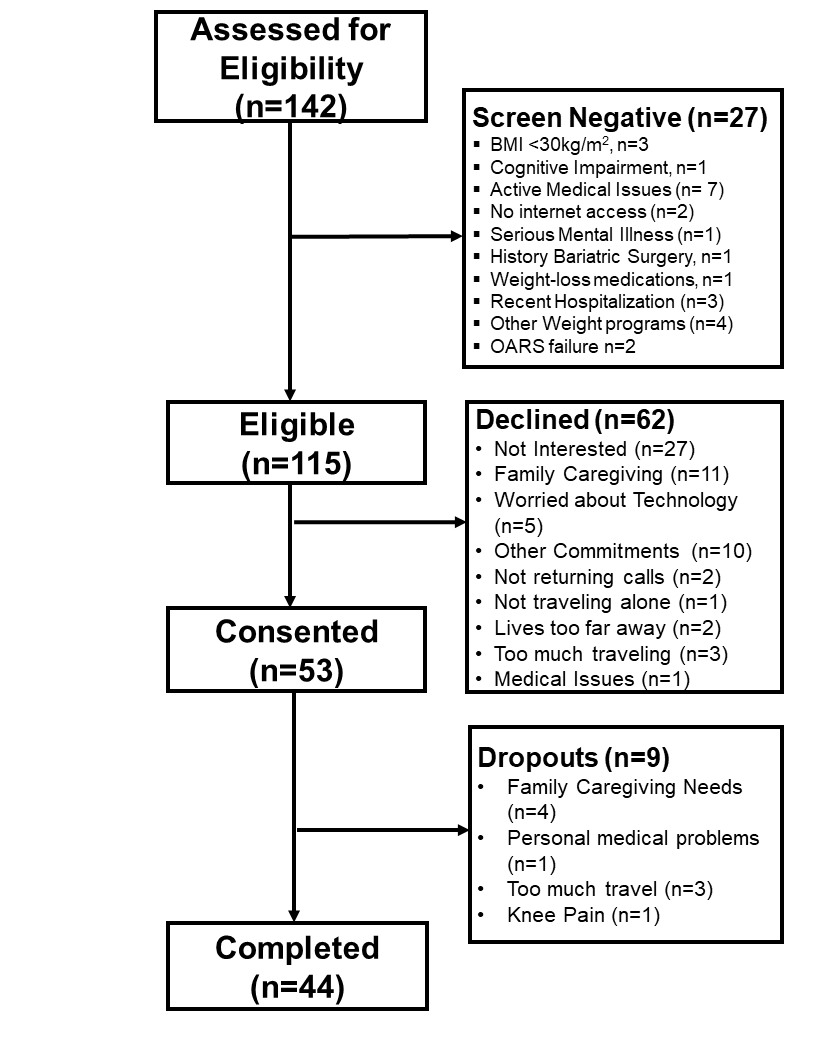

Supplement: Supplementary file 2 — Additional file 2. Appendix 2: Consort Diagram of Participant Flow [file 12877_2020_1978_MOESM2_ESM.tif]
