# Supplementary material for: Feasibility and acceptability of a technology-based, rural weight management intervention in older adults with obesity
Source: BMC Geriatr. 2021 Jan 12;21:44. doi: 10.1186/s12877-020-01978-x (PMC7801868; doi:10.1186/s12877-020-01978-x)
Supplement: Supplementary file 3 — Additional file 3. Appendix 3: Adverse Events [file 12877_2020_1978_MOESM3_ESM.docx]

**Appendix 3: Preliminary Outcome Measures of Study Cohort of Technology-Based Weight Loss Intervention**

|  | **Baseline (N=53)** | **Week 8 (n=44)** | **Week 16 (n=44)** | **Week 26 (N=44)** | **p value** |
| --- | --- | --- | --- | --- | --- |
| **Anthropometric** |  |  |  |  |  |
| Weight, kg | 99.3±17.1 | 95.3±16.0 | 94.1±15.9 | 93.2±16.0 | <0.001 |
| BMI, kg/m^2^ | 36.6±5.0 | 35.6±5.1 | 35.0±5.3 | 34.7±5.4 | <0.001 |
| ^#^Waist circumference, cm | 115.8±12.7 | 113.4±12.6 | 112.6±13.0 | 112.8±11.9 | <0.001 |
| ^#^Waist to hip ratio | 0.926±0.076 | 0.926±0.079 | 0.928±0.747 | 0.935±0.075 | 0.46 |
| **Objective Measures** |  |  |  |  |  |
| 30-second Sit To Stand, repetitions | 13.4±5.5 | 15.7±5.6 | 15.8±5.6 | 16.7±5.9 | <0.001 |
| ^#^6 min walk test, m | 387.8±91.7 | 420.6±88.1 | 430.6±93.9 | 425.0±91.7 | <0.001 |
| ^#^Gait Speed, sec | 1.069±0.223 | 1.064±0.209 | 1.064±0.218 | 1.036±0.203 | 0.045 |
| ^#^Grip Strength, kg | 25.3±9.5 | 25.6±10.7 | 27.7±9.8 | 25.9±10.6 | 0.034 |
| **Subjective Measures** |  |  |  |  |  |
| Late-Life Functionality |  |  |  |  |  |
| Total | 59.8±8.4 | 61.4±8.7 | 61.7±9.1 | 63.2±9.3 | <0.001 |
| Upper extremity | 79.5±13.6 | 77.5±12.2 | 78.6±12.9 | 81.0±12.7 | 0.02 |
| Basic lower extremity | 72.8±12.7 | 75.5±13.4 | 76.9±14.8 | 79.3±14.6 | <0.001 |
| Advanced lower extremity | 48.3±13.2 | 52.0±12.9 | 51.4±13.8 | 53.3±14.5 | <0.001 |

^#^Incomplete objective data on adults unable to perform follow-up assessments
