# Supplementary material for: Feasibility and acceptability of a technology-based, rural weight management intervention in older adults with obesity
Source: BMC Geriatr. 2021 Jan 12;21:44. doi: 10.1186/s12877-020-01978-x (PMC7801868; doi:10.1186/s12877-020-01978-x)
Supplement: Supplementary file 4 — Additional file 4. Appendix 4: Preliminary Outcome Measures of All Participants. [file 12877_2020_1978_MOESM4_ESM.docx]

**APPENDIX #4: Adverse Events from the Technology-Based Intervention**

|  | **Mild** | **Moderate** | **Severe** |
| --- | --- | --- | --- |
| **Serious Adverse Events** | ER visit for atrial fibrillation (n=1) | **---** | **---** |
| **Possibly Related** | Musculoskeletal (n=21) | Musculoskeletal (n=2) | **---** |
|  | Neck Pain (n=2) | Back pain (n=1 | **---** |
|  | Shoulder Pain (n=6) | Knee Pain (n=1) | **---** |
|  | Hand Pain (n=2) |  |  |
|  | Leg Pain (n=2) |  |  |
|  | Back Pain (n=5) | Constitutional (n=1) | **---** |
|  | Knee Pain (n=3) | Dizziness/Fatigue (n=1) | **---** |
|  | Heel Pain (n=1) | Psychiatric (n=1) | **---** |
|  |  | Depression (n=1) | **---** |
| **Definitely Related** | Musculoskeletal (n=6) | Endocrine (n=1)  Hypoglycemia (n=1) | **---** |
|  | Back Pain (n=1) |  |  |
|  | Knee Pain (n=3) |  |  |
|  | Upset stomach (Vitamin D [n=1]) |  |  |
|  | Rash with Fitbit (n=1) |  |  |
